# Method for selective quantification of adipose-derived stromal/stem cells in tissue

**DOI:** 10.14440/jbm.2016.127

**Published:** 2016-11-03

**Authors:** Akira Nishimura, Takeo Kumagai, Masaru Nakatani, Kotaro Yoshimura

**Affiliations:** ^1^Kaneka Corporation, Kobe MI R&D Center ^3^F 6-7-3, Minatojima, Minamimachi, Chuo-ku, Kobe, Hyogo 650-0047, Japan; ^2^Department of Plastic Surgery, School of Medicine, Jichi Medical University, 3311-1 Yakushiji, Shimotsuke, Tochigi 329-0498, Japan

**Keywords:** adipose-derived stromal/stem cells, adipose tissue, ASC isolation technique, fat graft, selective quantification

## Abstract

Fat grafts are valuable for soft-tissue regeneration and augmentation. However, fat graft systems require further improvement for the prediction of graft retention. The concentration of adipose-derived stromal/stem cells (ASCs) is one of the most important factors that affect graft retention; however, current cell quantification techniques have not been applied to adipose tissue. Here we developed a method for the selective quantification of ASCs in tissue (SQAT). We identified a characteristic methylated site in the CD31 promoter after searching for specific markers of ASCs. This DNA methylation was not detected in any cell type other than ASCs in adipose tissue. Therefore, analyzing this methylation may be a suitable approach for quantifying ASCs in tissues because DNA is readily extracted from tissues. SQAT is based on quantifying this methylation by quantitative polymerase chain reaction using methylation-sensitive HapII-treated DNA as the template. SQAT was validated based on the numbers of ASCs determined by CD31^−^/CD34^+^-based flow cytometry. The results obtained by both methods were perfectly correlated, thereby demonstrating that SQAT is a useful tool for quantifying ASCs. SQAT analysis using ASCs isolated from suctioned fat according to the standard protocol (*i.e.*, collagenase treatment) showed that the yield of ASCs was 59% ± 21%, which suggests that the ASC isolation technique requires further improvement. Furthermore, SQAT is an excellent method for quantifying ASCs in arbitrary samples (particularly tissue), which could dramatically improve ASC isolation technologies and fat graft systems, thereby facilitating the prediction of graft retention.

## INTRODUCTION

Autologous fat grafts have attracted increasing attention in the field of soft-tissue regeneration and augmentation because fat grafts have advantages such as simple operation, low costs, low rejection rate, soft feel, and a natural look [1, 2]. However, the fat graft system requires further improvement for the prediction of graft retention. It has been reported that fat graft retention rates considerably vary from 20% to 80%, resulting in unpredictable outcomes [3-5].

Adipose-derived stromal/stem cells (ASCs) comprise one of the most important factors associated with graft retention. Several studies suggested that ASCs function during the healing and remodeling of transplanted fat by secretion of growth factors (*e.g.*, human growth factor, transforming growth factor β, and vascular endothelial growth factor) and differentiation [6-8]. Yoshimura *et al.* [9, 10] reported that the cotransplantation of isolated ASCs can result in long-term retention of the graft, which is termed cell-assisted lipotransfer. Philips *et al.* [11] found that the number of ASCs isolated from adipose tissue by collagenase treatment was highly proportional to graft retention, suggesting that the concentration of ASCs in transplant fat is one of the biological factors that can predict fat graft retention. However, predictions of retention have been impractical because the current ASC quantitative techniques have not been directly applied to adipose tissue.

Currently, the quantification of ASCs is based on the analysis of characteristic protein expression profiles, such as CD31^−^/CD34^+^ [11-13]. Flow cytometry (FCM) is the most common method for quantification; however, it has been applied only to cell suspensions and not tissues. Although imaging analysis based on microscopy is a possible approach, this form of visual analysis lacks objectivity and cannot be quantitative.

CD31 (platelet endothelial cell adhesion molecule-1) is found on the surfaces of endothelial cells where it exhibits adhesive properties [14], and ASCs are well known to possess the CD31– immunophenotype [11-13]. The CD31 promoter contains no TATA or CAAT elements, but it has an initial element that is commonly found in TATA-less promoters [15], which indicates that DNA methylation is associated with the inhibition of CD31 expression [16, 17]. Philippe *et al.* [18, 19] found that ASCs have a highly methylated CD31 promoter.

Here we developed a method for the selective quantification of ASCs in t issue (SQAT), which is based on the quantification of the methylation site of interest in the CD31 promoter by quantitative polymerase chain reaction (qPCR). To the best of our knowledge, this is the first study reporting direct/selective quantitative analysis of ASCs in tissues, and it may facilitate the prediction of graft retention.

## MATERIALS AND METHODS

### Ethics statement and human tissue sampling

This study was approved by the ethics committees at Kaneka Corporation (No. 2015-03) and Cellport Clinic Yokohama (15^th^ Ethics Committee). Human subcutaneous lipoaspirates were collected as waste materials from five donors following elective surgery at Cellport Clinic Yokohama after obtaining their informed consent. The characteristics of the donors are shown in **Table S1**. The mean age was 40.6 years (range, 28–60 years), and the mean body mass index was 19.5 kg/m^2^ (range, 15.8–21.2 kg/m^2^).

Human umbilical vein endothelial cells (HUVECs) (P1), human pericytes from placenta (hPC-PLs) (P2), and human mononuclear cells from peripheral blood (hMNC-PBs) were purchased from PromoCell (Heidelberg, Germany).

### Stromal vascular fraction preparation

The stromal vascular fraction (SVF) was prepared from human subcutaneous lipoaspirate according to previously described methods with some modifications [20, 21]. The processed lipoaspirate was digested with shaking at 37°C for 30 min using an equal volume of 0.1% collagenase (Cell Dispersion, Wako, Osaka, Japan) in Hanks’ Balanced Salt solution (Sigma, St Louis, MO, USA). Subsequently, the suspension was centrifuged to collect the SVF pellet. SVF was washed three times and passed through a 100- µ m mesh filter (Corning, NY, USA).

### Preparation of adipose-derived stromal/stem cells and adipocytes

To purify ASCs, SVF (1.5–2.0 × 10^7^ cells) was plated onto a 150-mm culture dish and cultivated in minimum essential medium alpha (containing nucleosides) supplemented with 10% fetal bovine serum (HyClone, USA)/antibiotic-antimycotic (Gibco, Massachusetts, USA) at 37°C and 5% CO_2_ under humid conditions. After culture for 72 h, the nonadherent cells in the dish were completely removed with phosphate-buffered saline (Gibco), and the adherent cells were cultured to subconfluence. Concentrated ASCs (adherent cells) were collected using TrypLE Select (Gibco).

To prepare adipocytes, ASCs were induced to adipogenic differentiation using a StemPro Adipogenesis Differentiation Kit (Gibco). The confluent culture of ASCs was incubated under adipogenic conditions for 14 d. After detachment by TrypLE Select (Gibco), floating cells (adipocytes) were separated from the precipitate (immature cells) by centrifugation, and the adipocytes were collected using micropipettes.

### DNA extraction

DNA was extracted from each sample using a NucleoSpin Tissue kit (Macherey-Nagel, Düren, Germany), according to the manufacturer’s instruction. Adipose tissues were homogenized with a BioMasher II (Nippi, Tokyo, Japan) and used as samples.

### Bisulfite sequencing analysis

Bisulfite modification of the purified DNA was performed using a MethylEasy Xceed Rapid DNA Bisulphite Modification Kit (Human Genetic Signatures, North Ryde NSW, Australia) according to the manufacturer’s instructions. The converted DNA was amplified by polymerase chain reaction (PCR) with TaKaRa EpiTaq HS (Takarabio, Shiga, Japan), Bisulfite PCR Fw, and Bisulfite PCR Rv. The following PCR protocol was used: 98°C for 3 min and 35 cycles each at 98°C for 10 s, 55°C for 15 s, and 72°C for 1 min. The PCR products were purified with NucleoSpin Gel and a PCR Clean-up kit (Takara) and sequenced using Bisulfite sequence Rv. The primers used in this study are listed in **Table S2**.

### HapII digestion

The purified DNA was fragmented by passing it through a 27-gauge needle and digested with 5 units/ml HapII (Takara) at 37°C. After 2 h, the enzyme reaction was terminated by heating at 80°C for 20 min.

### Quantitative polymerase chain reaction

PCR was performed with the StepOnePlus Real-Time PCR System (Applied Biosystems, Massachusetts, USA). The total volume in each reaction was 20 μ l, which comprised 10 μl of twofold SYBR Premix Ex Taq GC, 5 μl of HapII-treated DNA, and 0.5 μM of each primer (C_6th_ Fw and C_6th_ Rv, as shown in **Table S2**). The following PCR protocol was used: 95°C for 30 s followed by 40 cycles each at 95°C for 15 s and 60°C for 30 s, and then one cycle at 95°C for 15 s, 60°C for 30 s, and 95°C for 15 s. All samples were analyzed in triplicate.

### Percentage DNA methylation

Ct values obtained for the HapII-treated template were individually subtracted from the Ct values for the untreated template (reference) to obtain the ΔCt value. The methylation percentage was calculated as follows: methylation percentage (%) = 100 × 2^−ΔCt^.

### Flow cytometry analysis of the ASC count in SVFs

The total cell numbers were determined using a NucleoCounter NC-100 (Chemometec, Allerød, Denmark). SVF was analyzed by FCM to determine the ratio of ASCs to SVF. Cells in SVF were incubated with FITC-conjugated CD31 (BD Biosciences, New Jersey, USA) and PE-conjugated CD34 (BD Biosciences). The labeled cells were washed with phosphate-buffered saline and analyzed using a FACSCalibur flow cytometer (CellQuest software, BD Biosciences). ASCs were defined as the CD31^−^/CD34^+^ subpopulation [11-13]. ASCs in SVF were calculated as follows: ASCs in SVF (cells) = total cell number × ASC:SVF.

## RESULTS

### Development of selective quantification of ASCs in tissue

**Figure 1:**
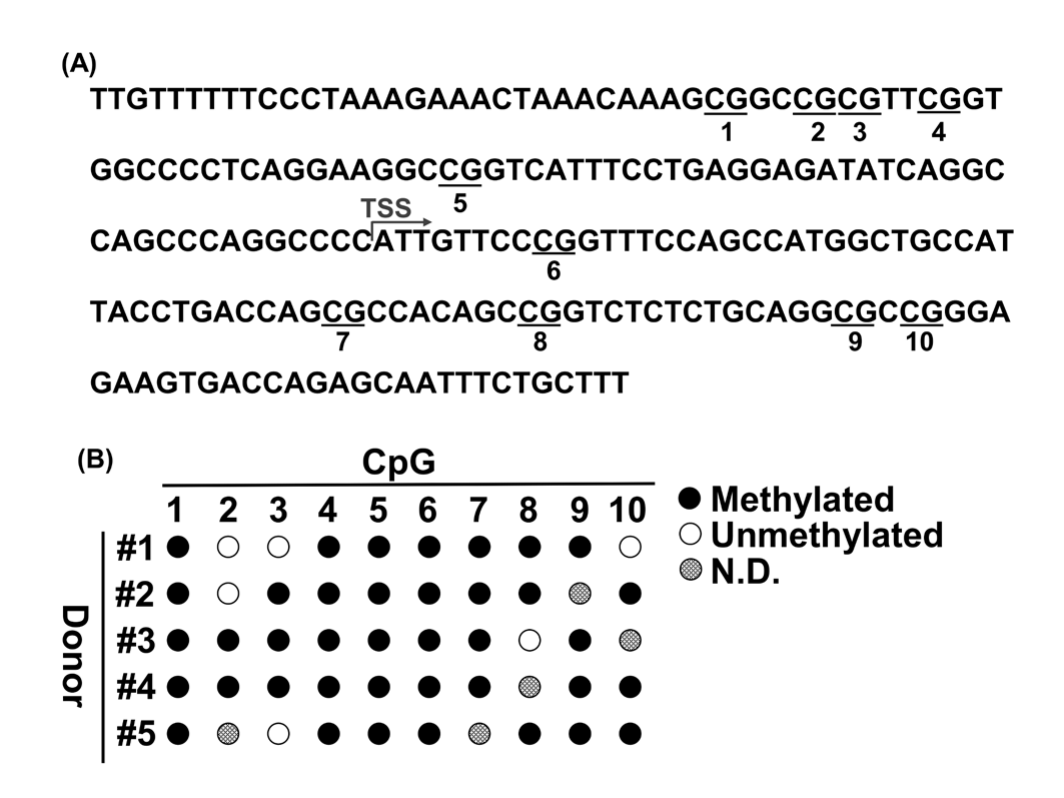
**CpG methylation in the CD31 promoter in ASCs**. **A**. Distribution of CpGs in the CD31 promoter. CpG sites are underlined and numbered from the 5'-most CpG starting with 1. **B**. Bisulfite analysis of the CD31 promoter in ASCs. Methylated CpGs, unmethylated CpG and ND are indicated by closed circles, open circles, and dotted circles, respectively. TSS, transcription start site; ND, not determined.

To determine whether CpG methylation in the CD31 promoter is a marker of ASCs, we analyzed the CpG methylation levels in the CD31 promoter in ASCs (5 donors) by bisulfite sequencing. The CD31 promoter includes 10 potentially methylated sites in CpGs from −100 to +100 of the transcription start site [18] (**Fig. 1A**). As demonstrated in previous studies [18, 19], the CD31 promoter was found to be highly methylated in ASCs (**Fig. 1B**).

**Figure 2:**
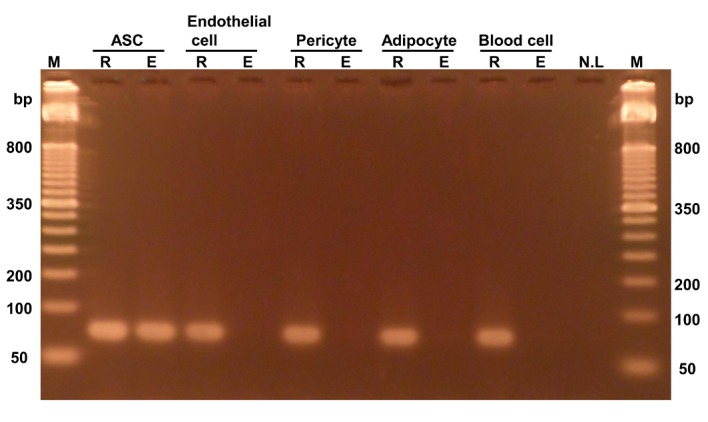
**Selective PCR amplification from ASCs with methylated C_6th_**. Genomic DNA extracted from each cell type was treated with either HapII (indicated by E) or no enzyme (indicated by R) and then used for PCR (25 cycles). HUVECs and hPC-PLs were used as surrogates for endothelial cells and pericytes, respectively. M, marker. NL, no load.

In particular, we found that the C_1st_, C_4th_, C_5th_, and C_6th_ sites were stably methylated in all donors, which indicates that methylation of these sites could be a valuable marker for the identification of ASCs. Hence, we tested whether the methylation of C_6th_ specifically occurs only in ASCs using qPCR because adipose tissue comprises various cell types, including mostly ASCs, endothelial cells, pericytes, adipocytes, and blood cells [22]. Each PCR product was analyzed using gel electrophoresis to confirm the specific amplification of the samples (**Fig. 2**). Under the condition with no enzyme, PCR products were detected from all samples. Under the conditions with HapII enzyme treatment, the amplification product was clearly detected in ASCs, whereas the amplification products were not detected in any other cell types except for ASCs. Therefore, we measured the methylation percentage for the C_6th_ site in each cell type (**Table 1**). As shown in **Figure 2**, ASCs had a high methylation level (almost 100%), whereas the other cell types had low methylation levels (< 4%). These results strongly suggested that methylated C_6th_ is a valuable marker of ASCs and that ASCs can be quantified using methylated C_6th_ as an index.

**Figure 3:**
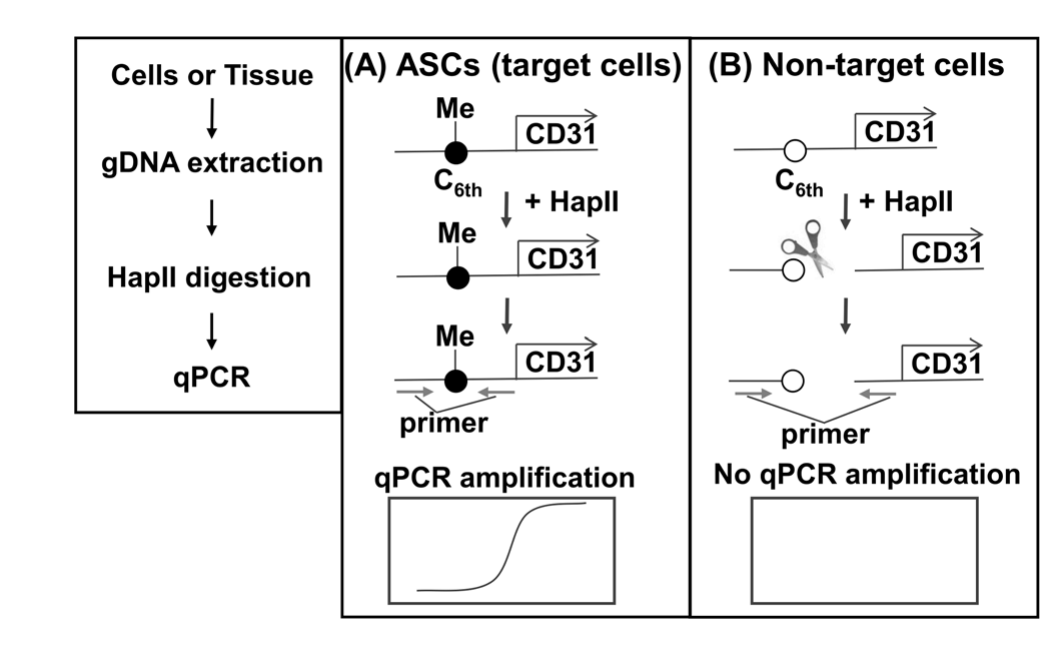
**Principle of the selective quantification of ASCs in tissue (SQAT) method**. Genomic DNA from samples is treated by HapII (a methylation-sensitive restriction enzyme) and then used as the template for the qPCR reaction. **A**. In ASCs, the sixth CpG (C6th) (see **Fig. 1**) in the CD31 promoter is methylated and thus HapII-digestion is blocked completely, thereby allowing qPCR amplification. **B**. In non-target cells (*e.g.*, endothelial cells, pericytes, blood cells, and adipocytes), the unmethylated 6^th^ CpG (C_6th_) in the CD31 promoter in non-target cells is digested fully by HapII, thereby preventing qPCR amplification.

We developed the SQAT method, which comprises DNA extraction, methylation-sensitive restriction enzyme-HapII digestion, and qPCR (**Fig. 3**), to ensure simplicity and sensitivity. HapII specifically recognizes and cleaves the DNA sequence CCGG, which is present around C_6th_ (as shown in **Fig. 1A**) and its activity is clearly blocked by methylation. When the C_6th_ site is methylated (in case of ASC), HapII digestion is blocked, resulting in PCR amplification. In contrast, when the C_6th_ site is unmethylated (in all cell types except for ASCs), HapII digestion occurs, resulting in no PCR amplification.

To determine the sensitivity of SQAT, we generated a standard curve using serial dilutions (10^3^–10^7^) of ASCs (**Fig. S1**). Similar profiles were obtained with artificial samples containing HUVECs, hPC-PLs, and hMNC-PBs (ASCs:HUVECs:hPC-PLs:hMNC-PBs = 1:1:1:1) (data not shown). The standard curve obtained using SQAT indicated linear amplification and high sensitivity. To validate the SQAT method, we quantified ASCs in SVF using both SQAT and FCM (**Fig. S2**, **Table 2**). Results obtained using both methods were highly correlated; therefore, SQAT may be useful for quantifying ASCs.

### Quantification of ASCs in suctioned fat using selective quantification of ASCs in tissue

Limited information is available regarding the yield of ASCs from suctioned fat using the standard protocol (collagenase treatment). Here we determined the yield of ASCs from suctioned fat using the standard protocol with SQAT (**Table 3**). It was surprising that low yields of ASCs were obtained (59% ± 21%) and that the variation in the yield was high, which indicates that the ASC isolation technique can be improved further.

**Table 1:** Percentage of methylation at 6^th^ CpG (C_6th_) site in each cell type.

Cell type	Methylation (%) of the C_6th_ site^a^
ASC	100 ± 1
Endothelial cell	3 ± 1
Pericyte	2 ± 1
Blood cell	4 ± 3
Adipocyte	4 ± 5

^a^The values represent the means and standard deviations based on at least three independent experiments.

**Table 2:** Comparison of SQAT and flow cytometry for quantifying ASCs in SVF^a^.

Donor	SQAT (× 10^5^/1 ml PLA^b^)	FCM (× 10^5^/1 ml PLA^b^)	SQAT/FCM
# 1	1.9	2.3	0.8
# 2	1.5	1.3	1.2
# 3	3.8	3.6	1.1
# 4	1.7	1.9	0.9
# 5	2.7	2.0	1.4
Mean ± S.D.	-	-	1.1 ± 0.2

^a^The cell numbers represent averages based on three replicates. ^b^PLA, processed lipoaspirate.

**Table 3:** Yield of ASCs from suctioned fat using the standard protocol (collagenase treatment)^a^.

Donor	Suctioned fat (× 10^5^/1 ml PLA^b^)	SVF (× 10^5^/1 ml PLA^b^)	Yield (%)
# 1	2.1	1.9	90
# 2	4.3	1.5	35
# 3	6.5	3.8	58
# 4	3.7	1.7	46
# 5	4.1	2.7	66
Mean ± S.D.	-	-	59 ± 21

^a^The cell numbers represent averages based on three replicates. ^b^PLA, processed lipoaspirate.

## DISCUSSION

ASCs are well known to be of value in various clinical procedures, such as fat grafting, cartilage regeneration, and growth factor delivery [23]. Thus, there is a major demand for a procedure that enables the simple, selective, and sensitive quantification of ASCs in samples, including tissue samples. Several methods can be used for the quantification of ASCs (**Table 4**), which are based on analyzing characteristic protein expression profiles using FCM or image analysis. FCM has a high capacity for quantification; however, it has low sensitivity (> 2–3 g of adipose tissue required), and it cannot be applied to tissue samples. In contrast, imaging analysis involving microscopy can be applied to tissue samples; however, this method based on visual determination has a low capacity for quantification. Here we identified an epigenetic biomarker for ASC and developed SQAT, which has a high capacity for quantification, high sensitivity (10–20 μg as adipose tissue), and applicability to tissue samples. Our method can predict all cell types with the type-specific epigenetic DNA status, and quantitative methods involving epigenetic biomarkers are expected to be widely used in the near future.

**Table 4:** Comparison of quantitative methods for ASC.

Method	Sample limit (cells)	Quantitative	Applicability to tissue samples	Life and death decision
SQAT	< 10^2^ to 10^3^	+	+	−
Flow cytometry	< 10^5^ to 10^6^	+	−	+
Image analysis	< 10^2^ to 10^3^	−	+	+

SQAT has a disadvantage because it cannot determine the cell viability because the DNA extraction process kills all cells, whereas FCM and image analysis can readily determine the cell viability. Ethidium monoazide (EMA) may overcome this limitation because EMA is a photoactivated molecule that covalently binds to DNA under light conditions, and it can only enter dead cells with damaged cell membranes [24]. DNA that covalently binds to EMA cannot be amplified by PCR [25]. Therefore, after pretreatment using EMA before DNA extraction, dead cells can be excluded and only live cells will be detected by SQAT. Currently, we are attempting to determine suitable conditions for pretreatment of samples with EMA, particularly tissue samples.

Fat grafts are valuable for soft-tissue regeneration and augmentation; however, to date, graft retention cannot be predicted. However, previous studies have indicated that fat graft retention may be predicted by quantifying the abundance of ASCs in transplant fat [9-11]. SQAT can quantify ASCs in transplant fat, which may facilitate the prediction of graft retention. Thus, we propose a future system for fat grafting, as follows. First, a standard curve must be prepared based on the correlation between graft retention and the number of ASCs in transplant fat quantified by SQAT. Further, ASCs in a small amount of fat obtained by presuction can be quantified by SQAT and the fat graft retention probability in each patient may be predicted from the standard curve before an operation. Based on the predicted retention, the doctor and patient will select an appropriate volume of transplant fat to improve the expected outcomes.

The results of the present study indicate that the conventional isolation technique for ASCs based on collagenase is inefficient and that uncollected ASCs have been discarded (**Table 3**). This is disadvantageous because ASCs are useful tools for regenerative therapy, similar to mesenchymal stem cells obtained from alternative sources [26-28]. Our unpublished results show that uncollected ASCs are present in the undigested residue after collagenase treatment. Currently, we are aiming to improve the isolation technique for ASCs using additional enzymes and chemicals.

In conclusion, we reported that SQAT has a high capacity for quantification and is applicable to tissue samples. Therefore, SQAT can improve the fat graft system and help to predict graft retention in the future.

## Abbreviation used

ASCs: adipose-derived stromal/stem cells
EMA: ethidium monoazide
FCM: flow cytometry
hMNC-PBs: human mononuclear cells from peripheral blood
hPC-PLs: human pericytes from placenta
HUVECs: human umbilical vein endothelial cells
PCR: polymerase chain reaction
qPCR: quantitative PCR
SQAT: selective quantification of ASCs in tissue
SVF: stromal vascular fraction

## Supplementary information

**Figure S1**. Standard curve obtained for ASCs using the selective quantification of ASCs in tissue (SQAT) method.

**Figure S2**. Flow cytometry analysis of the ASC count in SVF from each donor.

**Table S1**. Donor characteristics.

**Table S2**. Primers used in this study.
